# Timing of cochlear implantation in large vestibular aqueduct syndrome–a retrospective cohort analysis

**DOI:** 10.3389/fneur.2025.1562198

**Published:** 2025-03-21

**Authors:** Xiao Liu, Wanting Huang, Yunxiu Wang, Jingjing Xu, Lulu Xie, Lin Liu, Jia Chen

**Affiliations:** ^1^Department of Otorhinolaryngology, Second Affiliated Hospital, School of Medicine, Zhejiang University, Hangzhou, China; ^2^Department of Radiology, Second Affiliated Hospital, School of Medicine, Zhejiang University, Hangzhou, China

**Keywords:** cochlear implantation, large vestibular aqueduct syndrome, enlarged vestibular aqueduct, intervention criteria, decision-making, newborn hearing screening, residual hearing

## Abstract

**Introduction:**

Large vestibular aqueduct syndrome (LVAS) typically manifests fluctuating, progressive, or sudden hearing loss. Cochlear implantation (CI) is a critical intervention for LVAS patients when hearing aids (HA) no longer confer sufficient benefit. However, determining the optimal timing for CI remains challenging due to the heterogeneous and unpredictable nature of hearing loss progression, particularly when audiological criteria for CI are met, and HA can still provide benefits. This study aimed to address these complexities by analyzing real-world data on the timing of CI and clinical decision-making processes in pediatric LVAS patients.

**Methods:**

This retrospective cohort study reviewed the medical records of 74 pediatric patients (<18 years) with LVAS who underwent CI at a tertiary care hospital in China between 2010 and 2023. Clinical data, including newborn hearing screening (NBHS) results, methods of hearing loss identification, hearing levels at the initial audiological assessment (IAA), and patterns of hearing loss progression, were analyzed. Additionally, key milestones were evaluated, including age at hearing loss identification, IAA, and CI, and the durations between these events.

**Results:**

The median age at CI was 4.9 years (IQR: 3.0–6.8), with a median duration from IAA to CI of 2.9 years (IQR: 1.6–5.2). Patients identified through NBHS underwent CI earlier than those identified through poor response to sound or language learning difficulties. Moreover, patients with poor performance at IAA had an earlier age at CI and shorter duration from IAA to CI. CI timing was comparable among different hearing loss progression patterns. Finally, among patients meeting CI criteria but still benefiting from HA, while those who directly underwent CI had an earlier age at implantation, their interval from IAA to CI was similar to those who initially underwent HA fitting.

**Conclusion:**

The majority of LVAS patients experience progressive hearing loss and undergo CI during early childhood. Failure of NBHS and poor auditory performance at IAA are indicative of rapid hearing deterioration. Once audiological criteria for CI are met, prolonged observation appears unnecessary. Nevertheless, further prospective longitudinal studies are warranted to refine the timing and decision-making process.

## 1 Introduction

As is well documented, large vestibular aqueduct syndrome (LVAS), also referred to as hearing loss (HL) with enlarged vestibular aqueduct (EVA), is the most prevalent inner ear malformation (1, 2). Patients with LVAS typically present with fluctuating, progressive, and/or stepwise exacerbations of HL, whereas other patients may manifest congenital HL or experience sudden HL secondary to minor head trauma, shifts in barometric pressure, and the Valsalva maneuver (2). Cochlear implantation (CI) is considered the optimal treatment for patients with LVAS when conventional hearing aids (HA) can no longer confer benefits for language rehabilitation. According to earlier studies, early intervention could enhance language development and overall quality of life for patients with LVAS (3–6). However, given the complex and variable nature of hearing fluctuation patterns, it is challenging to determine the optimal timing for CI in patients with LVAS.

The clinical management of LVAS varies across countries and CI centers but generally begins with the identification of hearing impairment through newborn hearing screening (NBHS) or behavioral observations. Following identification, an initial audiological assessment (IAA) is conducted to determine whether the patient meets the criteria for CI. While some patients meet the audiological criteria for CI, they can still benefit from HA. Balancing the preservation of residual hearing with the need for timely intervention to optimize language outcomes presents a significant challenge. A waiting period, often spanning months to years and involving repeated audiological assessments, is employed to monitor hearing stability and evaluate the effectiveness of HA use (4, 7). However, if this observation period is prolonged or coincides with the critical period of language development, it can adversely affect speech and language acquisition (8). Additionally, the fluctuating and unpredictable nature of HL in LVAS, combined with variability in clinical practices and family preferences, further complicates the decision-making process.

Studies have explored the age at which CI is performed (5, 7, 9, 10). However, research examining the duration of hearing loss prior to CI is limited. This raises two key questions in managing HL in LVAS patients. Firstly, if a patient with LVAS presents with severe-profound HL at the IAA, is it appropriate to directly recommend CI, or should a trial with HA be initially recommended? Secondly, once a patient experiences hearing deterioration and meets the audiological criteria for CI, should a more aggressive approach toward CI be adopted, or should a conservative “wait-and-see” strategy be followed?

This study aimed to answer these two questions by retrospectively analyzing the medical records of LVAS patients who underwent CI and investigate key factors such as the age and method of HL identification, initial hearing levels, hearing fluctuations patterns, age at CI, and duration of HL before CI. The objective was to expand our understanding of the management of LVAS-related HL deterioration and provide evidence-based guidance for recommending CI.

## 2 Materials and methods

### 2.1 Design

This retrospective cohort study was a part of a clinical trial (ClinicalTrials.gov Identifier: NCT04934605) and approved by the Ethics Committee of the Second Affiliated Hospital Zhejiang University School of Medicine (SAHZU) in Hangzhou, Zhejiang, China (Approval No. 2024–0769). Data were collected from medical records of all pediatric patients (<18 years old at the time of CI) who underwent unilateral CI through the Cochlear Implant Program of the China Disabled Persons Federation (CDPF) at SAHZU over a 13-year period from 2010 to 2023.

### 2.2 Participants

A total of 74 patients with confirmed LVAS were included in this study. EVA was identified using temporal bone high-resolution computed tomography (HRCT) and/or inner ear magnetic resonance imaging (MRI). The diagnostic criteria for EVA were based on the standards established by Valvassori and Clemis (1) and defined as a vestibular aqueduct diameter > 1.5 mm at the midpoint and > 2 mm at the operculum on axial images. Other types of inner malformation were classified according to the criteria set by Sennaroglu and Saatci (11). Patients with incomplete partition type II (IP-II), characterized by vestibular dilation and the presence of only 1.5 cochlea turns accompanying EVA, were also included. The exclusion criteria were as follows: (1) Presence of syndromic deafness (except for Pendred Syndrome, which was included in this study). (2) Presence of other inner ear malformations (with the exception of IP-II, which was included in this study). (3) incomplete medical records.

### 2.3 Procedures

All participants in the Cochlear Implant Program of CDPF underwent a series of evaluations after the identification of HL to determine their eligibility for CI. These evaluations included audiological assessments, imaging studies, intellectual assessments, and evaluations of family and rehabilitation conditions. The criteria for CI candidacy were based on the Chinese “Guideline for Cochlear Implantation (2003)” (12) (applied to the patients who underwent IAA before 2014, *n* = 40) and the Chinese “Guideline for Cochlear Implantation (2013)” (13) (applied to the patients who underwent IAA from 2014 onward, *n* = 34).

An IAA was performed upon entry into the program to determine if patients met the audiological criteria for CI. The audiological candidacy criteria outlined in the 2003 and 2013 Chinese CI guidelines were broadly consistent, with minor adjustments in terminology and more explicit definitions of residual hearing. These criteria remained the same throughout the study period and were as follows: (1) Pre-lingual HL: short-tone auditory brainstem response (ABR) thresholds >90 dBnHL, auditory steady-state response (ASSR) thresholds >90 dBnHL at 2 kHz and higher frequencies, unaided behavioral audiogram thresholds >90 dBHL, or aided behavioral audiogram thresholds >50 dBHL at 2 kHz and higher frequencies. (2) Post-lingual HL: bilateral average thresholds in pure-tone audiometry >80 dBHL.

For patients who met the audiological criteria, a trial period of hearing aid use for 3–6 months was conducted. If the HA was ineffective or provided unsatisfactory hearing outcomes, CI was performed. If the HA trial was effective, patients’ families were given the option to proceed with CI (subject to scheduling) or continue HA use.

For patients with LVAS who did not initially meet the audiological criteria for CI, audiological assessments were repeated for at least 3 months following hearing deterioration. CI was considered only when hearing levels stabilized to meet the audiological criteria and HA use was no longer beneficial.

### 2.4 Data collection

Data were independently collected from medical records by two authors (X.L. and Y.W.), including demographic information (birth date and gender), medical history (HL identification, diagnosis details, type of hearing deterioration progression, past medical history, and personal history), imaging results (preoperative HRCT and/or inner ear MRI), audiological assessment results (preoperative ABR, ASSR, and audiogram), and CI surgery details (implantation date and side). Outcomes included age at CI, age at IAA, age at HL identification, and duration of HL.

### 2.5 Data analysis

The duration of HL before CI was defined as the difference between the age at CI and the age at HL identification. The duration before the assessment was defined as the difference between the age at IAA and the age at HL identification. The duration of the waiting time before CI was defined as the difference between the age at CI and the age at IAA.

Statistical analyses were performed using R v4.4.12024(R Inc., Boston, MA, United States). The Wilcoxon test (for two groups) or the Kruskal-Wallis test (for three or more groups) was used to compare differences in age at HL identification, age at IAA, age at CI, and the duration between time points across various methods of HL identification, initial hearing levels, and types of hearing deterioration progression. All reported *p*-values were two-tailed, and *p* < 0.05 was considered statistically significant.

## 3 Results

### 3.1 Clinical characteristics and timeline of cochlear implantation

**Figure 1:**
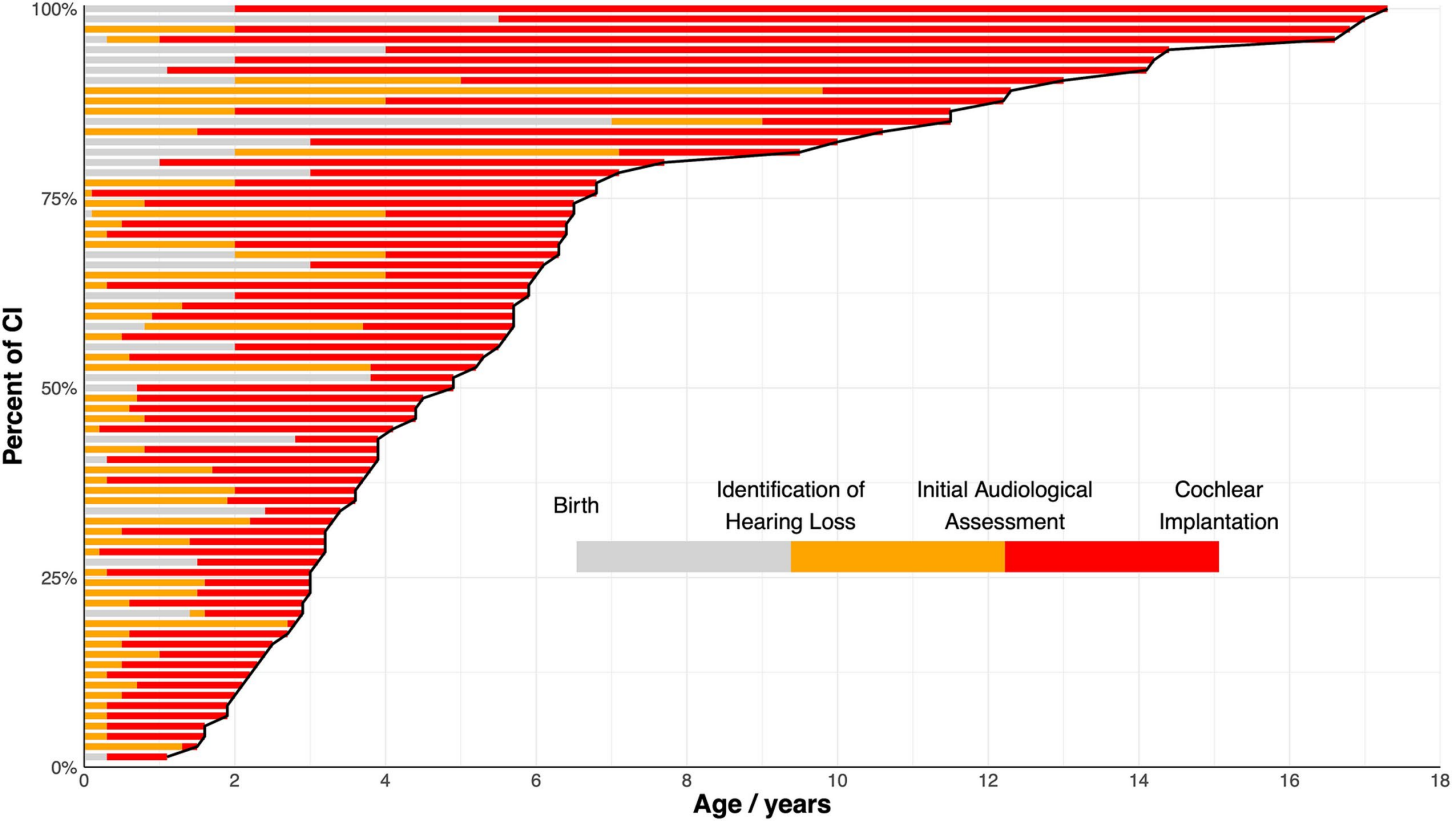
Percentage of cochlear implantation (CI) among the full cohort at different ages. Each bar represents an individual patient and is arranged by the age at CI. The varying shades within each bar represent the durations of different stages: from birth to identification of hearing loss (grey), from identification of hearing loss to initial audiological assessment (orange), and from initial audiological assessment to CI (red). The cure delineates the percentage of individuals undergoing CI as age increases.

The clinical characteristics of all 74 pediatric CI recipients with LVAS, comprising 41 boys and 34 girls are summarized in Table 1. All patients underwent unilateral CI. Sequential bilateral implantation was not performed in this cohort due to the guidelines of the Cochlear Implant Program of CDPF. A variety of CI devices were utilized, including Cochlear™ (CI24RE, CI512), MED-EL™ (C40, SONATA Ti100), Nurotron™ (CS-10A), and Advanced Bionics™ (HiRes 90 K). Standard surgical techniques were employed, including the round window approach or cochleostomy approach, with soft surgery principles applied to minimize intracochlear trauma. According to imaging results, 50 patients presented with isolated EVA, whereas the remaining 24 patients had EVA accompanied by IP-II. The distribution of CI recipients and their age at HL identification, IAA, and CI, organized by age at CI, are illustrated in Figure 1. A sensitivity analysis revealed no statistically significant differences between patients managed under the 2003 and 2013 guidelines in terms of duration between IAA and CI (median 3.3 years, IQR: 1.7–5.8 vs. median 2.7 years, IQR: 1.6–5.2; *p* = 0.5989).

**Table 1:** Clinical characteristics of 74 pediatric CI recipients with LVAS.

Sex, *n* (%)	
Male	41 (55.4%)
Female	34 (45.6%)
Inner ear malformation, *n* (%)	
Isolated EVA	50 (67.6%)
EVA + IP-II	24 (32.4%)
Newborn hearing screening, *n* (%)	
Screened	53 (71.6%)
Not screened	21 (28.4%)
Age at (years), median [interquartile] (range)	
Identification of hearing loss (ID)	0.0 [0.0, 1.2] (0.0, 7.0)
Initial audiological assessment (IAA)	1.4 [0.5, 2.5] (0.1, 9.8)
Cochlear implantation (CI)	4.9 [3.0, 6.8] (1.1, 17.3)
Duration (years), median [interquartile] (range)	
From ID to IAA	0.6 [0.1, 1.8] (0, 9.8)
From IAA to CI	2.9 [1.6, 5.2] (0.1, 15.6)
From ID to CI	4.1 [2.8, 6.4] (0.8, 16.8)
Type of implant, *n* (%)	
Cochlear CI24RE	39 (52.7%)
MED-EL C40	12 (16.2%)
MED-EL SONATA Ti100	8 (10.8%)
Nurotron CS-10A	8 (10.8%)
Cochlear CI512	6 (8.1%)
Advanced Bionics HiRes 90 K	1 (1.4%)
Surgical techniques, *n* (%)	
Cochleostomy	41 (55.4%)
Round window	33 (44.6%)

EVA, enlarged vestibular aqueduct; IP-II, incomplete partition type II.

### 3.2 Identification of hearing loss

**Figure 2:**
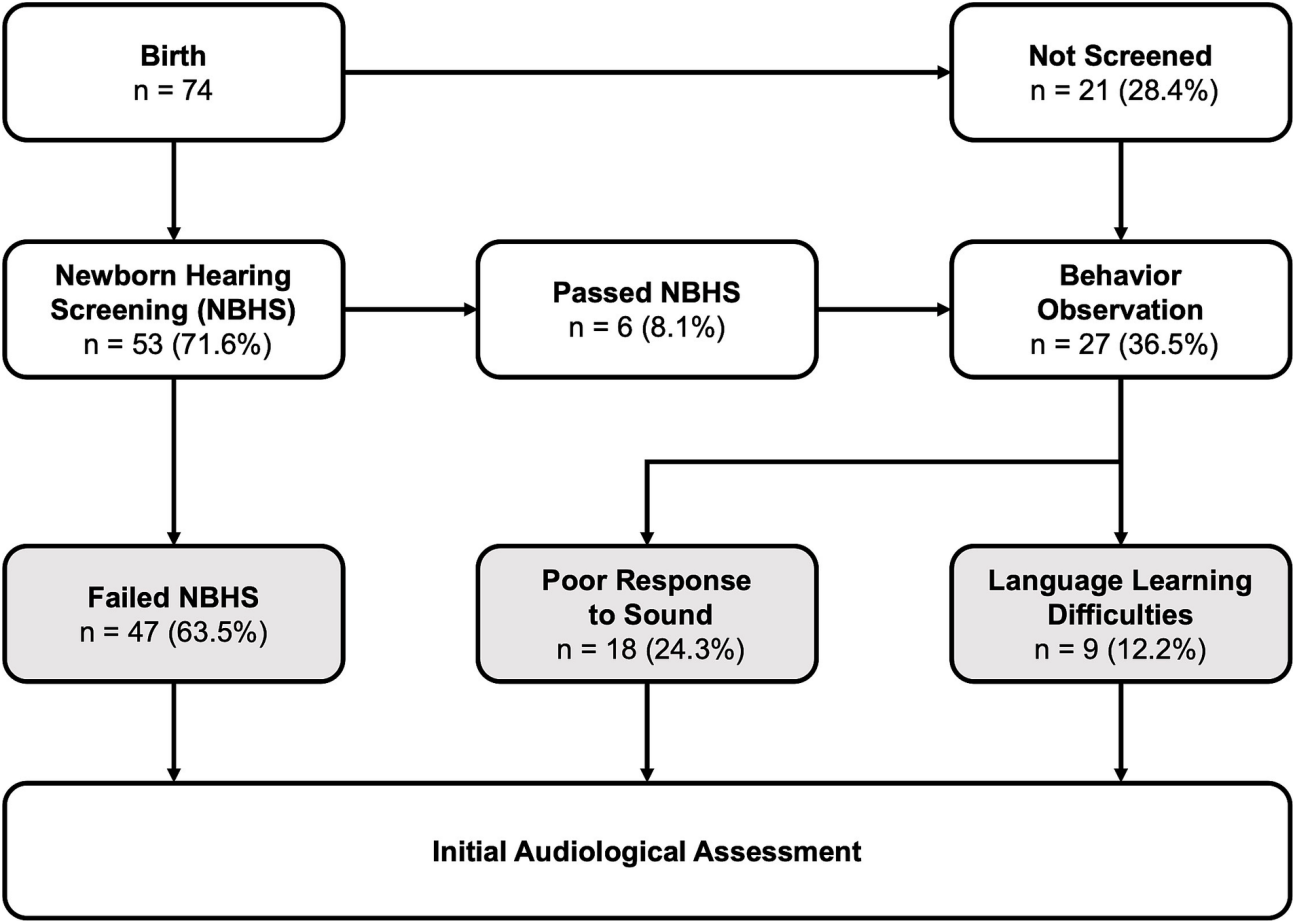
Flowchart of hearing loss identification outlining the three main methods of identifying hearing loss: failure in newborn hearing screening, poor response to sound (including self-reports), and language learning difficulties.

HL in LVAS patients was primarily identified via three methods (Figure 2), with the most common being the failure of NBHS, accounting for 63.5% (47/74) of all LVAS patients and 88.7% (47/53) of those who underwent screening. Only six patients (8.1% of all patients and 12.8% of screened patients) passed NBHS. Among patients who did not undergo NBHS, 36.5% (27/74) of the patients were identified during the behavioral observation stage. In the behavioral observation stage, HL was first identified in 66.7% (18/27 and 24.3% of all patients) due to poor response to sound (including self-reports) and in 33.3% (9/27 and 12.2% of all patients) due to language learning difficulties.

**Figure 3:**
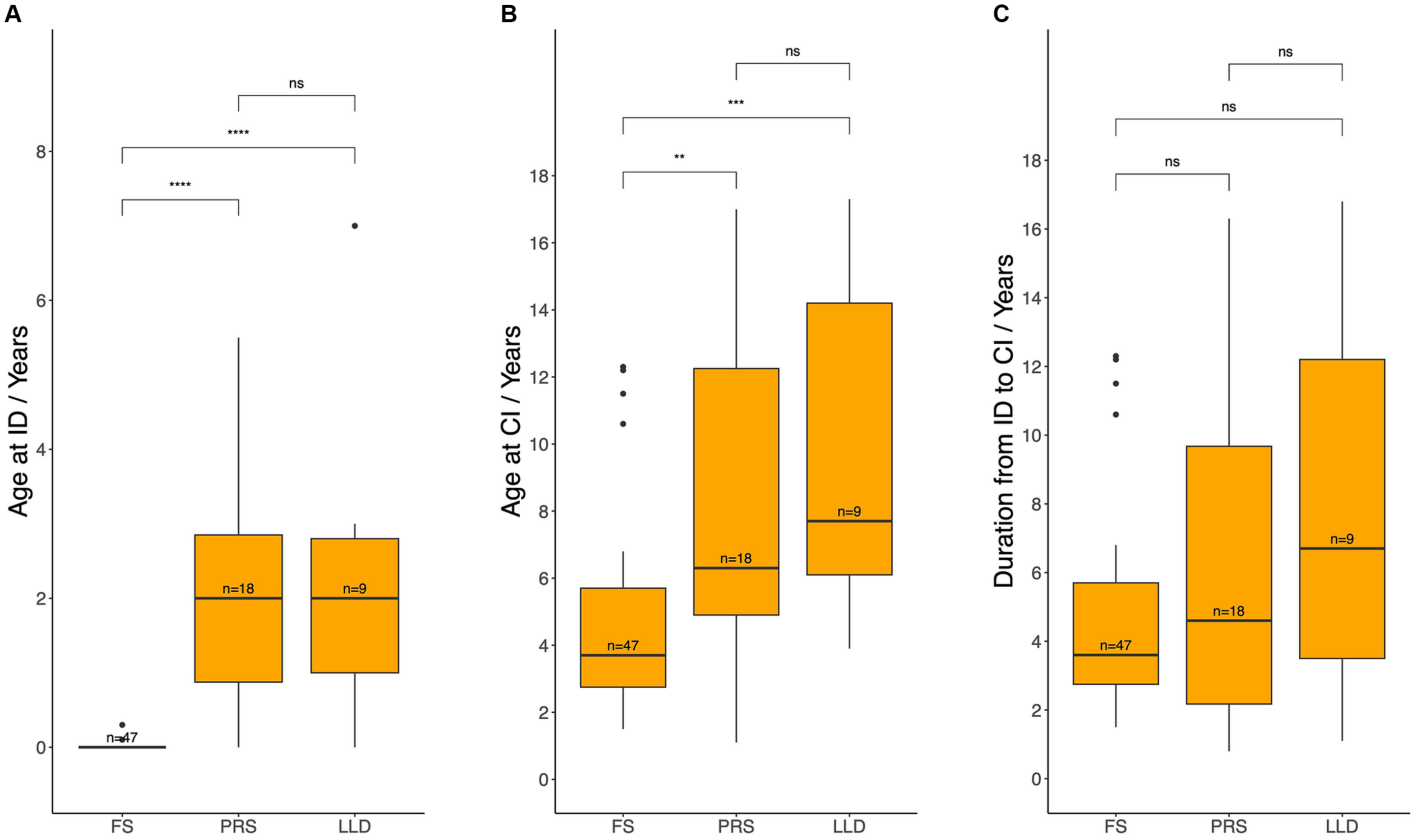
Age at hearing loss identification **(A)**, age at cochlear implantation **(B)**, and the duration between these two events **(C)** across different methods of identification. ID, hearing loss identification; CI, cochlear implantation; FS, failed newborn hearing screening; PRS, poor response to sound; LLD, language learning difficulties. Median age and durations between each pair of groups werecompared using the Wilcoxon test: ns = *p* > 0.05, ^**^p ≤ 0.01, ^***^p ≤ 0.001, ^****^p ≤ 0.0001.

Next, age at HL identification, age at CI, and duration from HL identification to CI were compared (Figure 3). As anticipated, HL was identified significantly earlier through failure of NBHS (median 0 years, IQR: 0–0) compared to behavioral observation due to poor response to sound (median 2.0 years, IQR: 0.9–2.9; *p* < 0.001) and language learning difficulties (median 2.0 years, IQR: 1.0–2.8; *p* < 0.001). Moreover, patients identified via NBHS underwent CI significantly earlier (median 3.7 years, IQR: 2.8–5.7) than those identified through poor response to sound (median 6.3 years, IQR: 4.9–12.2; *p* = 0.003) and language learning difficulties (median 7.7 years, IQR: 6.1–14.2; *p* < 0.001). However, the duration between identification and CI was similar between the groups.

### 3.3 Degree of hearing loss level at the initial audiological assessment

**Figure 4:**
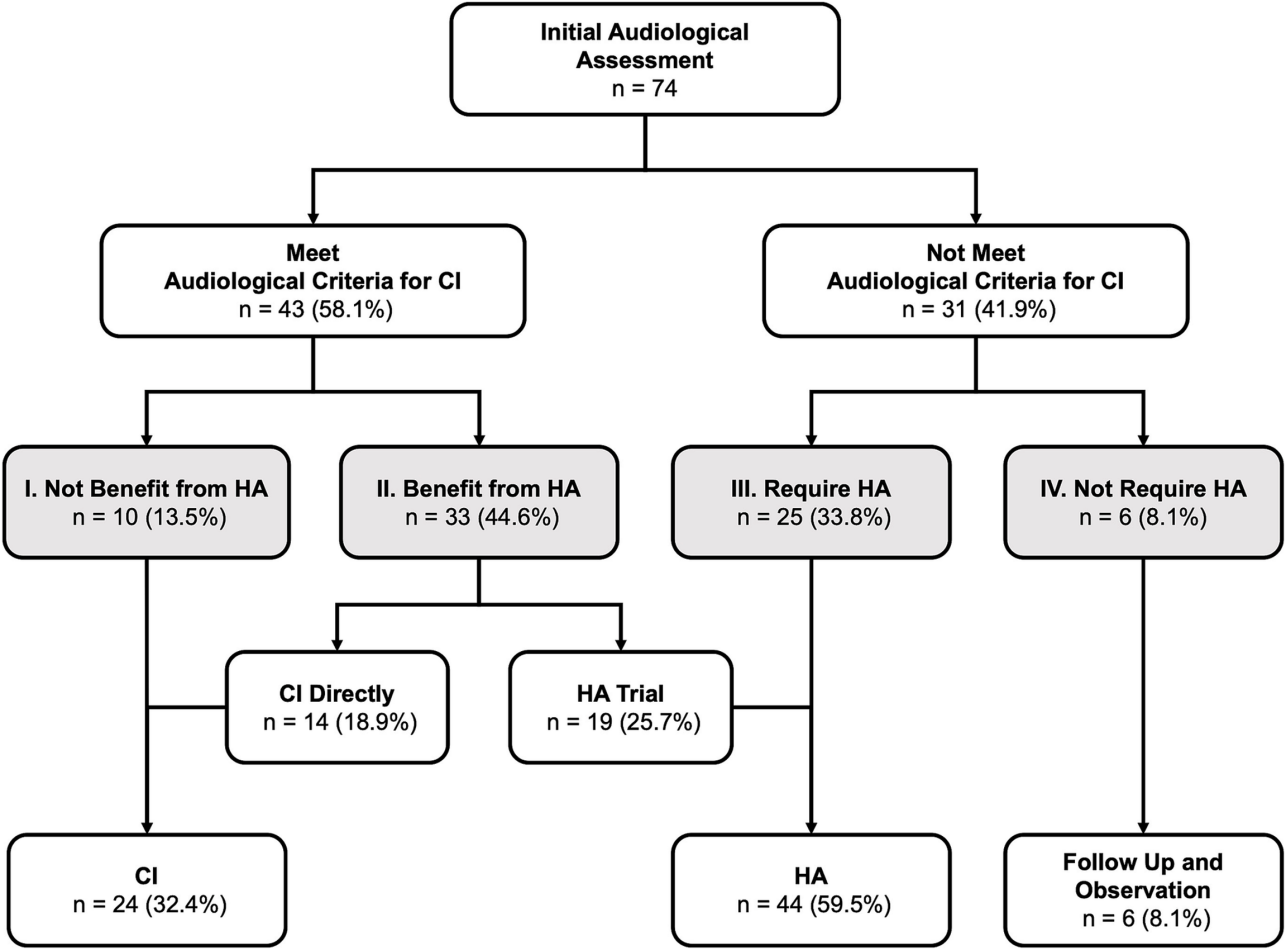
Results of initial audiological assessment and subsequent treatment: The degree of hearing loss was categorized into four levels, ranging from severe to mild: Level I (most severe) to Level IV (least severe). CI, cochlear implantation, HA, hearing aid(s).

After HL identification, all patients underwent IAA to confirm the diagnosis of HL and determine their eligibility for CI based on audiological candidacy criteria (Figure 4). In this cohort, over half of the patients (58.1%, *n* = 43) met the audiological criteria for CI at IAA. Among these candidates, only ten patients (23.3% of candidates and 13.5% of all patients) could not benefit from HA (Level I) and directly proceeded to CI. The majority of the candidates (*n* = 33, 76.7% of the candidates and 44.6% of all patients) could still benefit from HA (Level II). Among those who benefited from HA, 42.4% (*n* = 14, 18.9% of all patients) opted for CI surgery directly, while the remaining 57.6% (*n* = 19, 25.7% of all patients, 41.2% of candidates) were fitted with HA for language learning and/or rehabilitation. A total of 41.9% (*n* = 31) of patients did not fulfill the audiological candidacy criteria for CI. Among them, 80.6% (*n* = 25, 33.8% of all patients) required HA for language learning and/or rehabilitation (Level III), while 8.1% (*n* = 6) had sufficiently good hearing and did not require HA (Level IV).

**Figure 5:**
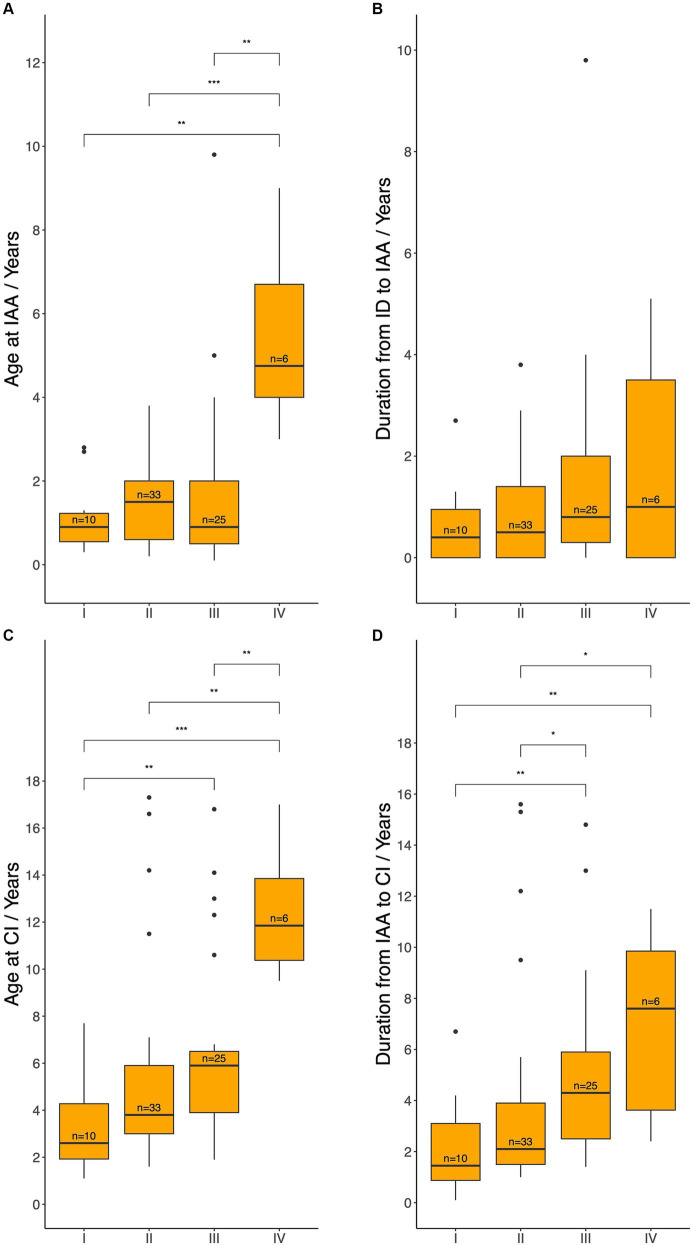
Age at initial audiological assessment **(A)**, duration from hearing loss identification to initial audiological assessment **(B)**, age at cochlear implantation **(C)**, and the duration from initial audiological assessment to cochlear implantation **(D)** across different hearing levels. IAA, initial audiological assessment; ID, hearing loss identification; CI, cochlear implantation; HA, hearing aids. Level I: met the audiological criteria for CI and could not benefit from HA, Level II: met the audiological criteria for CI but could benefit from HA, Level III: did not meet the audiological criteria for CI but required HA, Level IV: did not meet the audiological criteria and did not require HA. Median ages and durations between each pair of groups were compared using the Wilcoxon test: ns = *p* > 0.05 (not shown); _*_p ≤ 0.05; _**_p ≤ 0.01; _***_p ≤ 0.001.

Furthermore, age at IAA and CI, as well as the duration from HL identification to IAA and from IAA to CI were compared across different degrees of HL (Figure 5). Patients in Level IV underwent IAA significantly later than those in Levels I, II, and III [median 4.8 (IQR: 4.0–6.7) vs. 0.9 (IQR: 0.6–1.2), 1.5 (IQR: 0.6–2.0), and 0.9 (IQR: 0.5–2.0) years, respectively; *p* = 0.013, 0.002, 0.028]. No statistically significant differences were observed in the time between HL identification and IAA across levels. Regarding age at CI, patients with worse initial hearing levels underwent CI at a younger age. Specifically, patients in Level IV underwent CI significantly later than those in Levels I, II, and III [median 11.9 (IQR: 10.4–13.9) years vs. 2.6 (IQR: 1.9–4.3), 3.8 (IQR: 3.0–5.9), and 5.9 (IQR: 3.9–6.5) years; *p* = 0.014, 0.016, 0.055]. Likewise, patients in Level III underwent CI significantly later than those in Level I (*p* = 0.046). A similar trend was observed in the duration between IAA and CI. Indeed, the duration was significantly shorter for patients in Levels I (median 1.5 years, IQR: 0.9–3.1) and II (median 2.1 years, IQR: 1.5–3.9) compared to those in Levels III (median 4.3 years, IQR: 2.5–5.9) and IV (median 7.6 years, IQR: 3.6–9.9), with significant differences observed between Levels I and III (*p* = 0.0044), I and IV (*p* = 0.0108), II and III (*p* = 0.0218), and II and IV (*p* = 0.0338).

### 3.4 Hearing loss pattern prior to cochlear implantation

**Figure 6:**
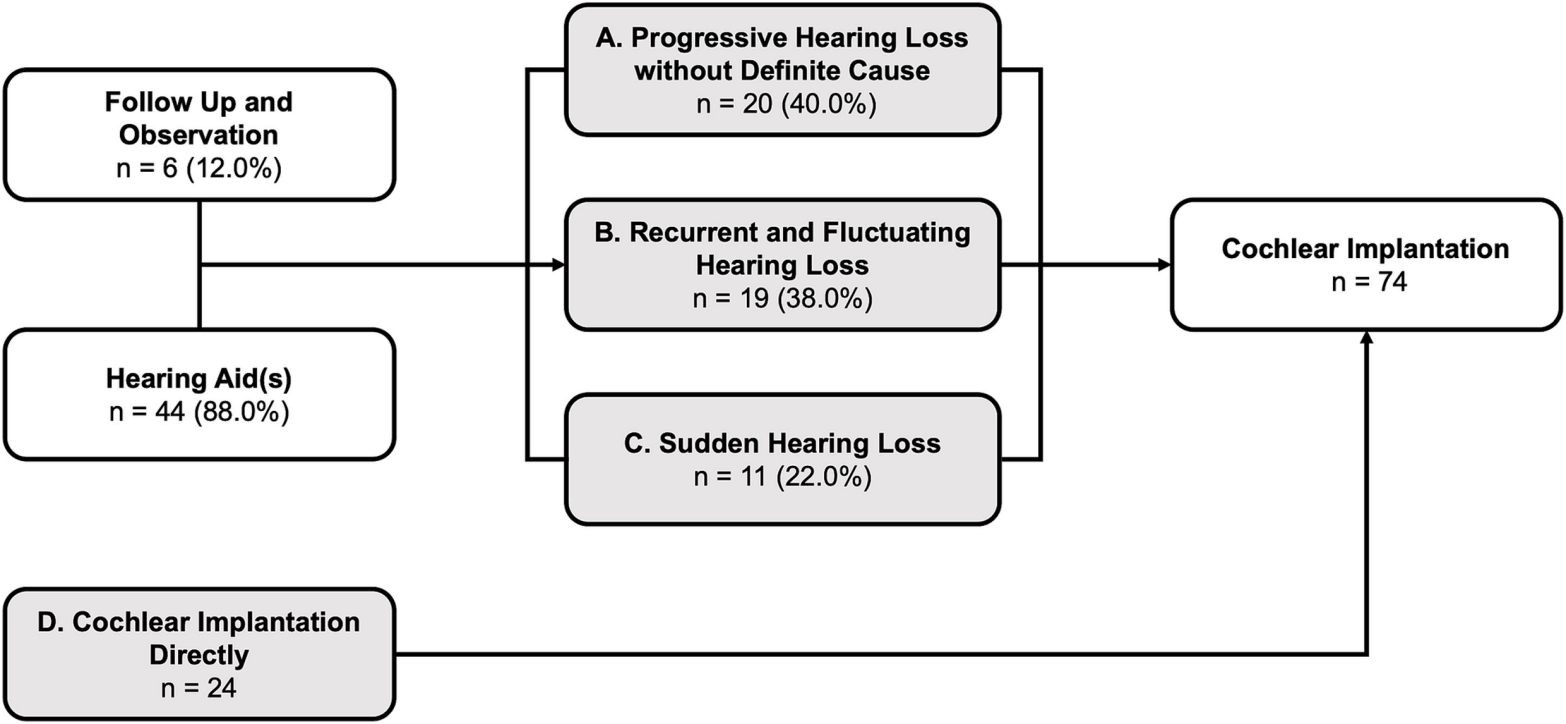
Patterns of hearing loss deterioration prior to cochlear implantation.

A total of 50 patients were observed and/or fitted with HA before eventually undergoing CI. The patterns of HL deterioration during this period are displayed in Figure 6. Among these patients, 40% (*n* = 20) experienced progressive HL that necessitated CI, without identifiable cause exacerbating the HL over time (Pattern A: Progressive). Another 38% (*n* = 19) exhibited various forms of recurrent fluctuating HL, characterized by gradual deterioration following multiple relapses, ultimately rendering HA ineffective and requiring CI (Pattern B: Fluctuating). The remaining 22% (*n* = 11) experienced sudden HL that did not recover within 3 months, eventually culminating in CI (Pattern C: Sudden). Additionally, the 24 patients who received CI directly without being fitted with HA were classified as Pattern D (Direct).

**Figure 7:**
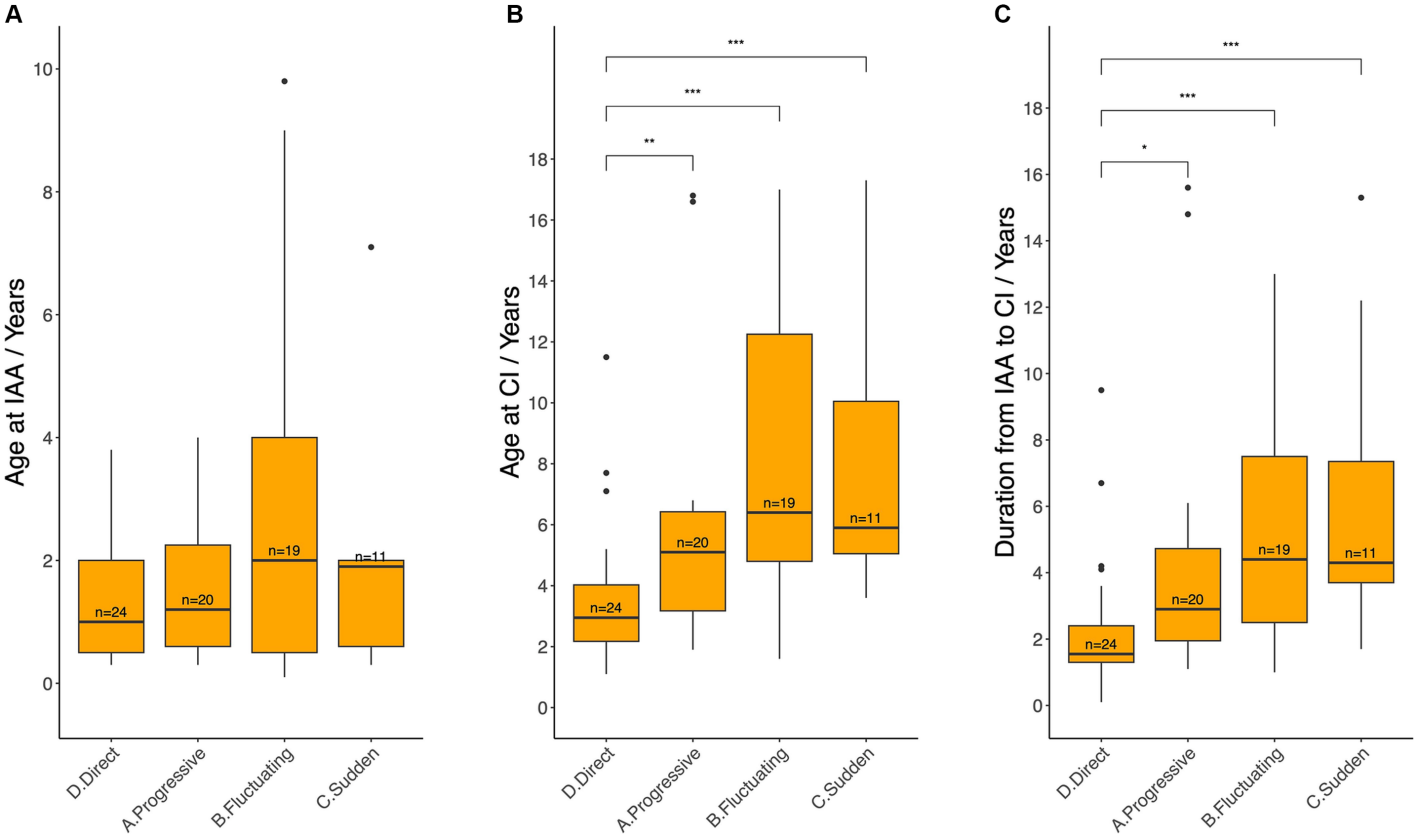
Age at initial audiological assessment **(A)**, age at cochlear implantation **(B)**, and the duration between these two events **(C)** across different hearing loss deterioration patterns. IAA, initial audiological assessment, CI, cochlear implantation. Median ages and durations between each pair of groups were compared using the Wilcoxon test: ns = *p* > 0.05 (not shown); ^*^ = *p* ≤ 0.05; ^**^p ≤ 0.01; ^***^p ≤ 0.001.

Age at IAA and CI, as well as the duration from IAA to CI were compared across different patterns of HL (Figure 7). Patients who underwent CI directly (Pattern D) were significantly younger at the time of CI (median 3.0 years, IQR: 2.2–4.0) and had a shorter duration from IAA to CI (median 1.6 years, IQR: 1.3–2.4) compared with patients in Pattern A (Age: 5.1 years, IQR: 3.2–6.4, *p* = 0.0089; Duration: 2.9 years, IQR: 2.0–4.7, *p* = 0.0101), Pattern B (Age: 6.4 years, IQR: 4.8–12.3, *p* = 0.0004; Duration: 4.4 years, IQR: 2.5–7.5, *p* = 0.009), and Pattern C (Age: 5.9 years, IQR: 5.1–10.1, *p* = 0.0085; Duration: 4.3 years, IQR: 3.7–7.3, *p* = 0.006). Conversely, no statistically significant differences were noted among Patterns A, B, and C in terms of age at CI or duration between IAA and CI. Similarly, no statistically significant differences were identified among Patterns A to D regarding the age at IAA.

**Figure 8:**
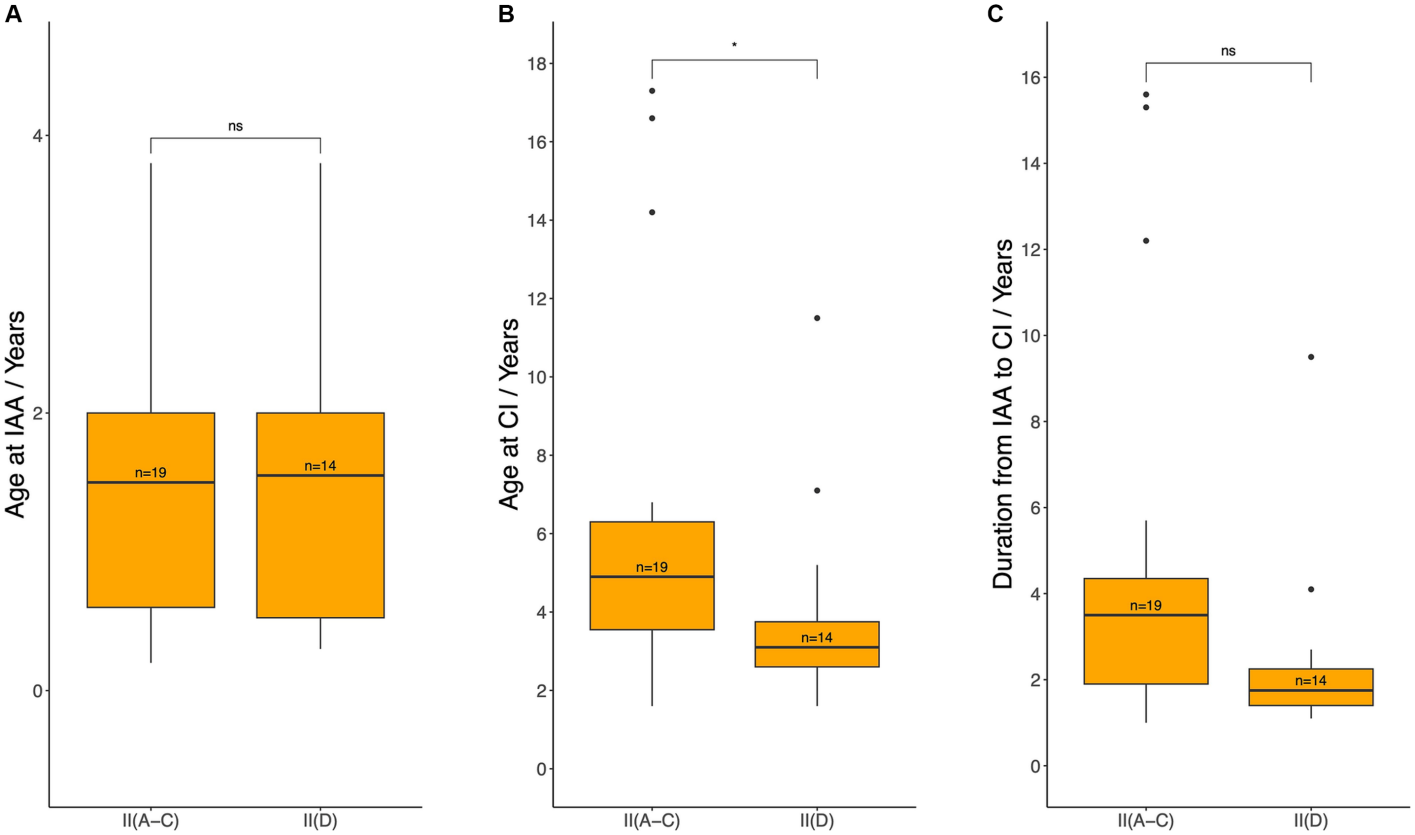
Age at initial audiological assessment **(A)**, age at cochlear implantation **(B)**, and the duration between these two events **(C)** for patients who met the audiological criteria for CI but could still benefit from hearing aids (Level II). IAA, initial audiological assessment, CI, cochlear implantation. Median ages and durations were compared between patients with Pattern D (group II (D), patients meeting CI criteria at IAA but opting for direct CI) and those with Patterns A–C (group II (A–C), patients meeting CI criteria at IAA but initially using hearing aids) using the Wilcoxon test: ns = *p* > 0.05; ^*^p ≤ 0.05.

Given that Level II patients could choose either HA fitting or direct CI, CI timing was compared between Pattern D and Patterns A, B, and C within Level II patients (Figure 8). Patients in Level II(D) had a statistically significantly younger age at CI (median 3.1 years, IQR: 2.6–3.8) compared to those in Level II(A-C) (median 4.9 years, IQR: 3.6–6.3; *p* = 0.0228). Although a shorter duration from IAA to CI was observed in Level II(D), the difference was not statistically significant [median 1.8 (IQR: 1.4–2.3) years vs. 3.5 (IQR: 1.9–4.4) years; *p* = 0.0556]. No statistically significant difference was noted between Levels II(D) and II(A-C) in the age at IAA [median 1.6 (IQR: 0.5–2.0) years vs. 1.5 (IQR: 0.6–2.0) years; *p* = 0.8834].

## 4 Discussion

Ascribed to the heterogeneous nature of HL in patients with LVAS (14, 15), CI timing and clinical decision-making are challenging. Generally, LVAS patients experience favorable CI outcomes, particularly those who undergo CI during infancy, often achieving comparable outcomes to infants with structurally normal inner ears (16). However, a relatively stable HL during the first few years after birth may lead to delayed CI, resulting in both verbal and nonverbal developmental delays (8). To maximize auditory and speech development outcomes and minimize the period without adequate auditory access, strict monitoring of hearing level and early CI are recommended (3–6, 17). This study provides valuable real-world data on CI timing and clinical decisions in LVAS patients.

Of note, patients with LVAS frequently experience long-term progressive HL and receive CI significantly later than those with typical congenital deafness (18). Herein, the median age at CI was 4.9 years, in line with the finding of a previous systematic review involving 55 studies that reported that the average age at CI in LVAS patients was 5.0 years (10). Furthermore, the proportion of patients who underwent CI sharply rose before 7 years old (78.4%) In agreement with these results, 74.5% of patients with LVAS met CI criteria before the age of 13 years (7). These findings collectively suggest that the majority of CI interventions were recommended and performed during early childhood, largely before the age of 7.

NBHS provides the earliest opportunity to detect HL in LVAS patients. Early counseling on CI in individuals with LVAS has been shown to reduce delays in implantation (6). In the present study, 63.5% of HL cases in LVAS were identified through NBHS, consistent with the 51.7% identified through NBHS in a previous study (5). Among the 53 screened patients only 12.8% passed NBHS, consistent with the observation of a previous report (19). We speculate that most of the remaining 21 patients (28.4%) who were not screened would likely have failed. Not undergoing NBHS could potentially lead to delayed diagnosis and intervention. However, another study reported that 45.9% of LVAS patients passed NBHS (20), a significantly higher proportion than observed in this study. Notably, most (79.8%) patients did not receive CI until an average age of 12.9 years when the data were collected (20). Taken together, these results indicate that passing NBHS does not necessarily correlate with stable hearing levels, whereas the failure of NBHS strongly suggests that earlier CI intervention may be necessitated.

Unlike other forms of congenital HL, patients with LVAS may meet CI criteria during periods of hearing deterioration, but often experience recovery shortly thereafter, making it difficult to finally determine the optimal timing for CI (17). Numerous factors can influence clinical decision-making for CI in LVAS, including medical factors such as genetics (17, 21, 22) and gender (5, 23), as well as socioeconomic considerations. Compared to other congenital hearing loss patients, those with LVAS are more likely to retain some residual hearing during audiological assessments. This preserved acoustic hearing, especially in low-frequency regions, has been shown to enhance speech-in-noise perception and music appreciation post-implantation (24–27). However, concern about losing remaining acoustic hearing is considered the most common reason for deferring CI, followed by economic considerations (7, 9). Even when patients meet CI criteria, a conservative “wait-and-see” approach is often adopted. In this study, more than half (58.1%) of patients with LVAS met the audiological criteria for CI at their initial hearing assessment. However, only 55.8% (24/43) of these candidates opted to directly proceed with CI. These results are consistent with those of other research that described that 59.5% of the ears met CI criteria at the initial visit, but nearly 40% of these ears did not receive CI before the age of 13 (7). This study specifically focused on a common but comprehensive clinical scenario: patients who meet CI criteria but could still benefit from HA (classified as hearing Level II in this study). Over half (57.6%, 19/33) of the Level II patients initially opted for HA (Level II (A-C)). Noteworthily, these patients underwent CI at an older age than those who chose CI directly (Level II (D)). Nevertheless, the difference in the duration from hearing assessment to CI was not statistically significant between Levels II (A-C) and II (D), implying that even when patients who meet CI criteria initially opt for HA, their hearing typically deteriorates rapidly, ultimately necessitating CI, with a timeline similar to those who directly proceed with CI.

In this study, 41.9% of patients with LVAS did not meet the audiological criteria for CI at IAA. All these patients underwent CI only after meeting the criteria, as the guideline standards were strictly adhered to. Over the past two decades, advancements in hearing preservation techniques (e.g., soft surgery, round window insertion) and electrode design have improved residual hearing retention rates, enhancing the feasibility of early CI for electro-acoustic stimulation (EAS) (22, 28). Given the inevitable progression of HL in LVAS, preemptive CI before meeting the criteria could be a viable option. However, this approach must be carefully weighed against the risks of unnecessary intervention, as fluctuating HL in LVAS may lead to temporary recovery, and even optimized surgical techniques cannot fully eliminate the possibility of residual hearing loss (28). Economic constraints and limited insurance coverage remain significant barriers to early CI in China (29), though the situation may vary in other countries. With ongoing economic development and the relatively decreasing cost of cochlear implant devices, this trend is likely to change. Therefore, even for patients expected to lose residual hearing, hearing preservation techniques should always be prioritized to maximize potential benefits.

Another challenge in decision-making for clinicians and families is the possibility of hearing recovery. CI centers may have varied experiences and strategies in managing these cases. In this study, patients were observed and followed up for at least 3 months after hearing deterioration to confirm the lack of improvement and the ineffectiveness of HA before undergoing CI. A review of patient records revealed that patients (or their families) who opted for directly undergoing CI (Pattern D) were younger age at CI and had a shorter duration from IAA to CI. However, no statistically significant differences were identified among those with progressive, fluctuating, or sudden HL (Patterns A, B, and C). Previous studies have developed diverse models for predicting LVAS HL progression. One model indicates a trend toward an 80 dB HL threshold during critical language acquisition years (3.2 to 6 years) regardless of hearing fluctuations (17). Another study, using language recognition rates such as Bamford-Kowal Bench scores, suggests CI at a recognition rate drop to 85% (3). Furthermore, a model based on vestibular aqueduct width indicates that each year after the first audiogram results in a 1.5 dB increase in speech recognition threshold and a 1.7% decline in word recognition score (30). Despite these findings, it is worthwhile acknowledging that most studies investigating prognostic factors for HL in LVAS have a high risk of bias due to limited adjustment for confounding factors (31). Closely following up and monitoring residual hearing are considered the optimal strategy (5, 7, 32).

This study was limited by its retrospective nature, with all data derived from patients who have undergone CI. Patients with stable hearing and who did not undergo CI surgery until adulthood were excluded, potentially shortening the observed duration of HL before CI and reducing the differences in CI age and duration among different HL patterns. Further prospective longitudinal cohort studies are warranted to more accurately and objectively describe the relationship between HL progression in LVAS patients and CI timing. Secondly, CI outcomes were not investigated in this study. The degree to which delayed cochlear implantation increases the risk of speech and language deficits in patients with LVAS remains elusive. Long-term follow-up studies exploring speech perception, language development, and quality of life outcomes in LVAS patients with varying CI timings are needed to address these uncertainties. Moreover, future studies should also evaluate the role of residual hearing preservation in these outcomes. Lastly, the study did not account for potential confounding factors such as socioeconomic status, access to healthcare, or variations in CI center practices, all of which may influence both the timing of CI and outcomes. Future studies should incorporate these factors to provide a more comprehensive understanding of decision-making and HL outcomes in patients with LVAS.

## 5 Conclusion

This study provides valuable insights into the timing of CI and clinical decision-making in patients with LVAS. While most LVAS patients experience progressive HL and undergo CI during early childhood, particularly before the age of 7, our findings highlight the need for early identification through NBHS, assessment of hearing levels at IAA, and close monitoring of residual hearing to limit developmental delays. Notably, our findings emphasize that even when patients who meet CI criteria initially opt for HA, their hearing often deteriorates swiftly, ultimately necessitating CI, with a timeline similar to those who directly proceed with CI. Overall, these findings indicate that no additional waiting is required for patients who meet CI criteria at IAA. Although prediction models have been proposed for HL progression, further prospective longitudinal cohort studies are needed to better define the optimal timing for CI and its impact on long-term outcomes.

## Data Availability

The raw data supporting the conclusions of this article will be made available by the authors, without undue reservation.
